# Activation of the Type I Interferon Pathway Is Enhanced in Response to Human Neuronal Differentiation

**DOI:** 10.1371/journal.pone.0058813

**Published:** 2013-03-07

**Authors:** Jocelyn R. Farmer, Kate M. Altschaefl, K. Sue O'Shea, David J. Miller

**Affiliations:** 1 Departments of Microbiology and Immunology, University of Michigan Medical School, Ann Arbor, Michigan, United States of America; 2 Department of Epidemiology, University of Michigan Medical School, Ann Arbor, Michigan, United States of America; 3 Department of Cell and Developmental Biology, University of Michigan Medical School, Ann Arbor, Michigan, United States of America; 4 Department of Internal Medicine, University of Michigan Medical School, Ann Arbor, Michigan, United States of America; University of Texas Medical Branch, United States of America

## Abstract

Despite the crucial role of innate immunity in preventing or controlling pathogen-induced damage in most, if not all, cell types, very little is known about the activity of this essential defense system in central nervous system neurons, especially in humans. In this report we use both an established neuronal cell line model and an embryonic stem cell-based system to examine human neuronal innate immunity and responses to neurotropic alphavirus infection in cultured cells. We demonstrate that neuronal differentiation is associated with increased expression of crucial type I interferon signaling pathway components, including interferon regulatory factor-9 and an interferon receptor heterodimer subunit, which results in enhanced interferon stimulation and subsequent heightened antiviral activity and cytoprotective responses against neurotropic alphaviruses such as western equine encephalitis virus. These results identify important differentiation-dependent changes in innate immune system function that control cell-autonomous neuronal responses. Furthermore, this work demonstrates the utility of human embryonic stem cell-derived cultures as a platform to study the interactions between innate immunity, virus infection, and pathogenesis in central nervous system neurons.

## Introduction

Neurons have historically been considered immunologically quiescent cells, but recent data suggest they can actively shape antiviral responses in the central nervous system (CNS). Neurons have functional viral pattern recognition receptor pathways [1], [2], [3], [4], produce innate immune cytokines such as type I interferons (IFNs) after viral infection [5], and respond to cytokine stimulation with cell-autonomous inhibition of virus replication and increased cell survival [6], [7], [8]. Innate immune responses mediated by type I IFNs are crucial for protection and recovery from CNS viral infections [7], [9], [10], and neurotropic viral pathogenesis is enhanced in mice with neural ectoderm-specific knockout of the type I IFN pathway [11]. These observations suggest that CNS-mediated control of virus replication, potentially via active neuronal innate immune pathways, is a critical component of host antiviral defenses. However, our knowledge of human neuronal innate immune function, and its impact on viral pathogenesis, is incomplete.

Arboviruses are the leading cause of viral encephalitis worldwide and represent prominent examples of emergent or resurgent pathogens with a significant impact on human health [12], [13], [14]. Arboviruses that target CNS neurons and produce encephalitis include bunyaviruses such as La Crosse virus, flaviviruses such as Japanese encephalitis virus, and alphaviruses such as western equine encephalitis viruses (WEEV). A frequently observed but poorly understood clinical characteristic of arboviral encephalitis is heightened disease severity in children, which may include the development of permanent post-infectious neurologic sequelae such as cognitive deficits, paralysis, and seizure disorders [14]. One hypothesis to explain this observation is that immature neurons or neural progenitor cells (NPCs), which are self-renewing multipotent precursors of astrocytes, oligodendrocytes, and neurons that are enriched in the developing CNS, have increased susceptibility to virus infection or viral-mediated damage compared to more mature neurons. Published experimental data support this hypothesis. Cultured neuronal cells display differentiation-dependent responses to viral infection, where undifferentiated cells have increased susceptibility to virus-mediated cell damage [6], [15], [16], [17], [18]. Furthermore, NPCs are permissive to neurotropic viral infections in vitro and in vivo, which can disrupt neurogenesis and differentiation [19], [20], [21], [22], [23], [24], [25], [26]. These observations suggest that intrinsic changes in cell-autonomous functions associated with neuronal development, such as innate immunity, may be important determinants in disease outcome.

We have previously demonstrated that human neurons derived from the BE(2)-C neuroblastoma cell line have differentiation-dependent responses to type I IFN stimulation [6]. In this report we investigated the underlying mechanism(s) responsible for this heightened responsiveness and found that BE(2)-C differentiation was accompanied by increased expression and function of central type I IFN pathway signaling components, most importantly one subunit of the type I IFN receptor heterodimer. Furthermore, we found that neurons derived from human embryonic stem cells (ESCs) displayed similar differentiation-dependent changes in innate immune system function and susceptibility to virus-induced damage.

## Materials and Methods

### Reagents

Tissue culture reagents were purchased from Invitrogen (Carlsbad, CA) with the following exceptions: brain-derived neurotropic factor (BDNF; Prospec, Rehovot, Israel), laminin and poly-D-lysine (Sigma, St. Louis, MO), and noggin (R&D Systems, Minneapolis, MN). Recombinant human IFNα-A/D, a hybrid universal type I IFN [27], was purchased from PBL Biomedical Laboratories (Piscataway, NJ) and stored as single use aliquots at −80^o^C.

Antibodies against the indicated targets were purchased as follows: NF200 (Sigma); neuronal nuclear antigen (NeuN) and poly-sialylated neural cell adhesion molecule (PSA-NCAM; Millipore, Billerica, MA); type I IFN receptor subunit 2 (IFNAR2; PBL Biomedical Laboratories); IFN regulatory factor (IRF)-7 (Cell Signaling Technology, Danvers, MA); IRF-9 (BD Transduction Laboratories, San Jose, CA); nestin (R&D Systems); major histocompatibility complex (MHC) class I (BioLegend, San Diego, CA); signal transducer and activator of transcription (STAT) 1, STAT2, phospho-STAT1, phospho-STAT2, Tyk2, Jak1, green fluorescent protein (GFP), and glyceraldehyde-3 phosphate dehydrogenase (GAPDH) (Santa Cruz Biotechnology, Santa Cruz, CA). We obtained antibodies against MxA from Otto Haller (University of Freiberg, Freiberg, Germany), and antibodies against Sox3 were generously provided by Michael Klymkowsky (University of Colorado at Boulder, Boulder, CO). All secondary reagents for immunoblot, immunocytochemistry, and flow cytometry analyses were purchased from Jackson Immunoresearch (West Grove, PA), except the Alexa Fluor® 488-conjugated streptavidin (Invitrogen).

The secreted alkaline phosphatase (SEAP) reporter plasmid driven by an IFN-stimulated response element (ISRE) promoter has been previously described [1], and the pTet-On plasmid was purchased from BD Biosciences (San Jose, CA). The overexpression plasmids encoding human IRF-9 and STAT2 were purchased from OriGene (Rockville, MD), and an overexpression plasmid encoding the human IFNAR2c isoform with a C-terminal hemagglutinin (HA) epitope tag (IFNAR2-HA) was generously provided by John Krolewski (University of California, Irvine, CA). IRF-9, STAT2, and IFNAR2-HA cDNAs were subcloned into pTRE2hyg (BD Biosciences) to achieve doxycycline-inducible expression. Cloning strategy details are available upon request.

### Viruses

Fort Morgan virus (FMV) strain CM4-146 was purchased from the American Type Culture Collection (Manassas, VA) and WEEV strain Cba-87 was generated from the full-length WEEV cDNA clone pWE2000 as previously described [6]. All experiments with infectious WEEV were done under Biosafety Level 3 (BSL-3) conditions in accordance with University of Michigan Institutional Biosafety Committee and CDC/NIH guidelines. All viruses were propagated in Vero cells and infectious virus titers in tissue culture supernatants were determined on Vero cell monolayers as previously described [6], where plaque assay sensitivity was 100 plaque-forming units (pfu)/ml. We infected cells with either FMV or WEEV at a multiplicity of infection (MOI) of 0.01 for all experiments with BE(2)-C neuronal cells. We harvested tissue culture supernatants for WEEV titers at 36 h post-infection (hpi), as previously published experiments indicated that 24 to 48 hpi was the optimal time frame to detect maximal differences between undifferentiated BE(2)-C and differentiated BE(2)-C/m cells with respect to both virus-induced cytotoxicity and virus production after type I IFN stimulation [6]. FMV is less virulent than WEEV, which results in delayed development of cytotoxicity in cultured cells at a similar inoculum. Preliminary experiments indicated that 72 hpi with an MOI of 0.01 was the optimal time point to measure virus production after type I IFN stimulation and separate FMV-induced cytotoxicity between undifferentiated BE(2)-C and differentiated BE(2)-C/m cells (data not shown).

To isolate purified virus for hESC-derived cell culture experiments, Vero cells were harvested at 48 hpi, tissue culture supernatants were centrifuged at 1,000× *g* for 5 min to pellet cellular debris, and virions were precipitated overnight by addition of polyethylene glycol and sodium chloride to 7% and 2.3% final concentrations, respectively. Virions were recovered by centrifugation at 3500× *g* for 20 min, resuspended in HBSS, loaded onto 15–45% linear sucrose step gradients, and centrifuged at 40,000× *g* for 90 min. Visible virion bands were collected, diluted in HBSS, pelleted at 35,000× *g* for 60 min, resuspended in HBSS, and stored at −80°C in single use aliquots.

### Cell Culture and Transfection

BE(2)-C human neuroblastoma cells were obtained from the American Type Culture Collection and were cultured and differentiated with all-*trans* retinoic acid as previously described [6]. Cells were transfected using Lipofectamine 2000 and stable cell lines were generated as previously described [1]. Conditional overexpression of IRF-9, IFNAR2, and STAT2 was induced by incubation with 1 μg/mL doxycycline for 36 h. We routinely obtained approximately 75% transfection efficiency in BE(2)-C cells, determined by in situ staining of cells transfected with either a control constitutive β-galactosidase expression vector or inducible IRF-9 or IFNAR2 overexpression vectors (data not shown). Furthermore, we included a constitutive GFP expression vector in co-transfection experiments to monitor transfection efficiency between experimental groups. Tissue culture SEAP levels were analyzed at 24 h post-induction with IFNα-A/D as previously described [1]. Cell viability after WEEV infection was determined using ATPlite (PerkinElmer, Waltham, MA) according to the manufacturer's instructions. This luminescence assay uses an ATP-dependent firefly luciferase to measure total cellular ATP levels after cell lysis, which provides a rapid and reproducible signal with long half-life, high sensitivity, and wide linear dynamic range.

The NIH-approved H7 hESC line was obtained from the WiCell Research Institute (Madison, WI) at passage 25. All hESC protocols were approved by the University of Michigan Human Pluripotent Stem Cell Research Oversight Committee. Undifferentiated H7 cells were maintained on feeder layers of irradiated mouse embryonic fibroblasts (GlobalStem, Rockville, MD) with daily changes of Dulbecco's modified Eagle's medium mixed 1∶1 with Ham's F12 (DMEM/F12) supplemented with 20% knockout serum replacement, 1 mM L-glutamine, 0.1 mM non-essential amino acids, 0.1 mM β-mercaptoethanol, and 4 ng/mL human basic fibroblast growth factor 2 (bFGF-2). hESCs were differentiated into NPCs in a noggin-independent manner using a modified protocol of previously published techniques [28]. In brief, H7 colonies were mechanically isolated from feeder layers and free-form differentiated in low attachment plates to produce cystic embryoid bodies. Embryoid bodies were harvested on day 4 and expanded for approximately three weeks on 0.1% gelatin in DMEM/F12 supplemented with 1% N2, 20 ng/mL bFGF-2, and 2 μg/mL heparin, and the resulting neuroepithelial rosettes were mechanically isolated and expanded into enriched populations of NPCs. Alternatively, hESCs were differentiated into NPCs in a noggin-dependent manner using a modified protocol of previously published techniques [29]. In brief, H7 colonies were mechanically isolated from feeder layers and transferred to low attachment plates in NPC media (DMEM/F12, 2% B27 supplement without vitamin A, 20 ng/mL bFGF-2) supplemented with 500 ng/mL noggin. After three weeks in suspension culture neurospheres were collected and triturated by pipette to smaller aggregates, plated on poly-D-lysine (50 μg/mL) and laminin (20 μg/mL) coated dishes, and allowed to expand as single cell cultures in NPC media. NPCs were subsequently differentiated for two weeks in NeurobasalTM media supplemented with 1% N2, 2% B27, 0.1 mM non-essential amino acids, and 10 ng/mL human BDNF.

### Immunoblot and RT-PCR

Whole cell lysates were harvested and analyzed by immunoblot as previously described [6]. Total RNA isolation and semi-quantitative RT-PCR were done as previously described [1], [30], and primer sequences are listed in Table 1. For quantitative RT-PCR (qRT-PCR), we generate cDNA from total RNA using iScript RT Supermix (Bio-Rad, Hercules, CA) with oligo dT and random hexamer primers according to the manufacturer's instructions. We completed PCR in triplicate samples using Sso Advanced SYBR Green Supermix (Bio-Rad) according to the manufacturer's instructions with a BioRad CFX96 Real Time thermal cycler and determined fluorescence threshold cycles (C_t_) with CFX96 Manager software. We normalized mRNA transcript levels to rRNA levels by calculating ΔC_t_ values of individual samples for statistical comparisons, and determined fold-increases using ΔΔC_t_ calculations.

**Table 1:** RT-PCR primer sequences.

Target	Primer ID	Sequence (5′–3′)
18S rRNA	hrRNA-F	CTTAGAGGGACAAGTGGCG
	hrRNA-R	ACGCTGAGCCAGTCAGTGTA
NGFR	hNGFR-F	ATCGGAGGGAATTGAGGTCT
	hNGFR-R	AATCCCCACAGGTCACAGTC
IFNAR1	hIFNAR1-F	TTGTGTGAAAGCCAGAGCAC
	hIFNAR1-R	TCAAGAAGACTTTCGCAGCA
IFNAR2	hIFNAR2-Tot-F	CACCAGAGTTTGAGATTGTTGG
	hIFNAR2-Tot-R^1^	GCTTGCTCATCACTGTGCTC
	hIFNAR2-Iso-R^2^	CACCATATCCATGGCTTCCA

1 Used for amplification of extracellular domain of IFNAR2 present in all three isoforms. Corresponds to primer indicated by black arrow in Fig. 2B.

2 Used for amplification of extracellular, transmembrane, and intracellular domains of IFNAR2. PCR product length differs in the three isoforms due to exon skipping and alternative splicing. Corresponds to primer indicated by grey arrows in Fig. 2B.

### Immunocytochemistry and Flow Cytometry

For immunocytochemistry analyses, cells were fixed in 2% paraformaldehyde, permeabilized in 0.1% Triton X-100, blocked in 10% goat serum, and incubated with primary antibody overnight at 4°C. The following day cells were sequentially incubated with Texas Red- or FITC-conjugated secondary antibodies and with 0.5 μg/mL 4,6-diamidino-2-phenyindole (DAPI) to stain nuclei. Cells were analyzed using an Olympus IX70 inverted microscope, final images were prepared using MetaMorph Premier Software, and all contrast adjustments to the final images were done prior to cropping. For flow cytometry analyses, cells were detached in 0.05% Trypsin-EDTA, filtered using 70 μm nylon mesh, and incubated with primary and corresponding fluorochrome-labeled secondary antibodies at 4°C. For intracellular staining, cells were fixed in 2% paraformaldehyde and permeabilized in 0.1% Triton X-100 at room temperature prior to antibody incubation. For IFNAR2 labeling, an additional amplification step was performed using a biotin-conjugated secondary antibody and Alexa Fluor® 488-conjugated streptavidin. A minimum of 10,000 cells were analyzed on a BD FACSCanto, and final histograms were assembled using FlowJo version 7.2.5. For extracellular staining, live cells were identified using 7-amino-actinomycin D exclusion.

### Statistics

Comparative statistical analyses were conducted using a two-tailed Student's *t* test where a *p* value of <0.05 was considered significant. For dose-titration analyses, EC_50_ and Hill slope values were calculated using Prism GraphPad 3.0. The EC_50_ represents the concentration of type I IFN that produced a half-maximal response. The Hill slope, or Hill coefficient, describes the fraction of receptor saturated by ligand as a function of the ligand concentration [31]. This parameter provides a quantitative measure of cooperative binding, where values >1 indicate positive cooperative binding, values <1 indicate negative cooperative binding, and values  = 1 indicate no cooperative binding.

## Results

### Human neuronal differentiation induces changes in type I IFN pathway signaling component expression and function

**Figure 1:**
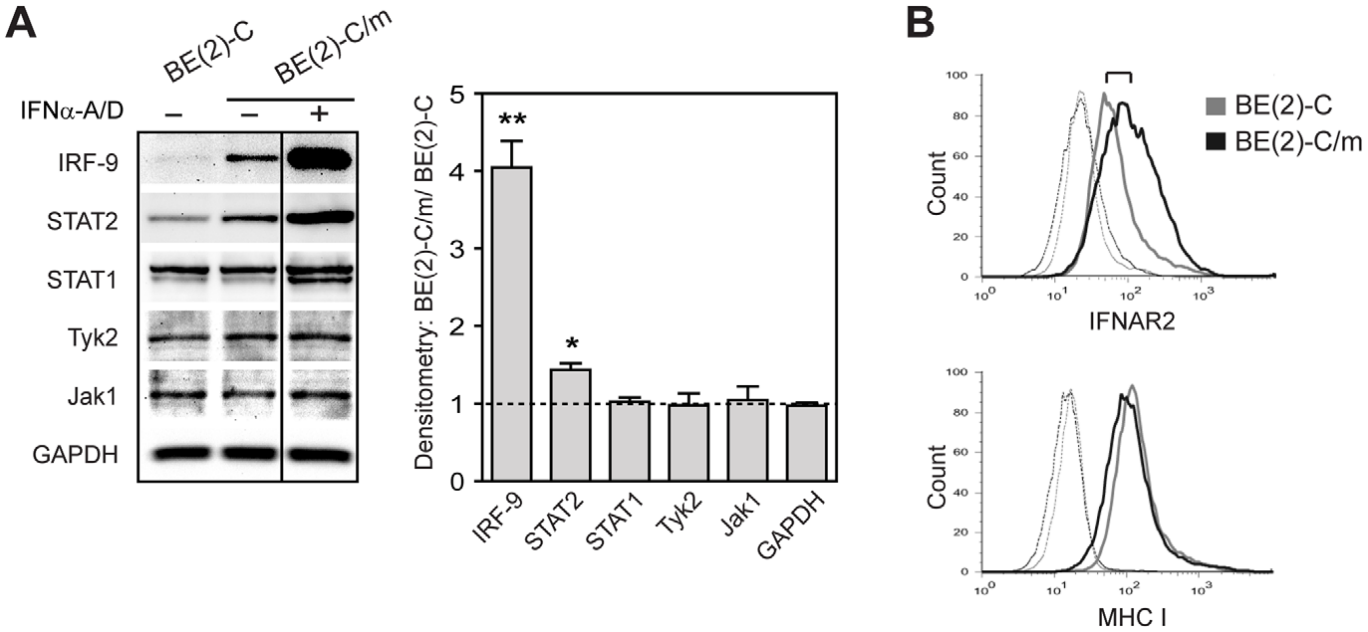
Differentiation-dependent changes in type I IFN pathway expression in human BE(2)-C neuronal cells. (A) Immunoblot analysis of the indicated type I IFN pathway component or GAPDH as a loading control. Lysate from IFNα-A/D-treated BE(2)-C/m cells was used as a positive control for the known IFN-inducible components IRF-9, STAT2, and STAT1. Representative blots from one of three independent experiments are shown, and quantitative immunoblot results shown on the graph represent mean ± SEM densitometry ratios of untreated differentiated over undifferentiated cells. *p<0.05, **p<0.005, relative to GAPDH. (B) Flow cytometry analysis of basal surface IFNAR2 expression in undifferentiated BE(2)-C cells (grey lines) and differentiated BE(2)-C/m cells (black lines). Dashed lines indicate background fluorescence levels obtained with isotype-matched control antibodies, and MHC class I surface expression was examined to assess potential global changes in cell surface protein expression. Representative overlaid histograms from one of five independent experiments are shown.

To investigate the underlying molecular mechanism(s) responsible for differentiation-dependent type I IFN responsiveness in human neuronal cells, we utilized a previously established culture system based on the neuroblastoma cell line BE(2)-C [6] and focused on canonical type I IFN signaling pathway components, including the surface receptor heterodimer composed of IFNAR1 and IFNAR2, the receptor-associated signal transduction kinases Jak1 and Tyk2, and the transcription factors IRF-9, STAT1, and STAT2. Initial genome-wide microarray analyses revealed upregulation of IFNAR2 and IRF-9, but not IFNAR1, Jak1, Tyk2, STAT1, or STAT2, in differentiated BE(2)-C/m cells [1] (microarray data available at www.ncbi.nlm.nih.gov/geo/under accession number GSE16452). We validated protein expression by immunoblot analysis and flow cytometry (Fig. 1). Differentiated BE(2)-C/m cells had a four-fold increase in IRF-9 and a one- to two-fold increase in STAT2 expression compared to undifferentiated cells, whereas there were no differences in STAT1, Tyk2, or Jak1 expression (Fig. 1A). We were unable to reliably detect either IFNAR subunit by immunoblot analysis or surface expression of IFNAR1 by flow cytometry (data not shown). However, flow cytometry did reveal a differentiation-dependent increase in IFNAR2 cell surface expression (Fig. 1B, upper histogram), where the quantitative ratio of IFNAR2 surface expression between differentiated and undifferentiated cells, determined by background-corrected median fluorescence intensity values and represented by the bracket in Fig. 1B, was 2.2±0.9 (*p*<0.05). This increase in IFNAR2 expression was not due to a global increase in surface protein levels in BE(2)-C/m cells, as the expression of MHC class I, which we used as a convenient surface protein to monitor global protein expression, was not significantly different between differentiated and undifferentiated cells (Fig. 1B, lower histogram). Although resting CNS neurons do not normally express MHC class I molecules on their surface, under appropriate stimulation conditions they are induced and may play a crucial role in development and synapse formation, in addition to their role as regulators of the adaptive immune response [32], [33].

**Figure 2:**
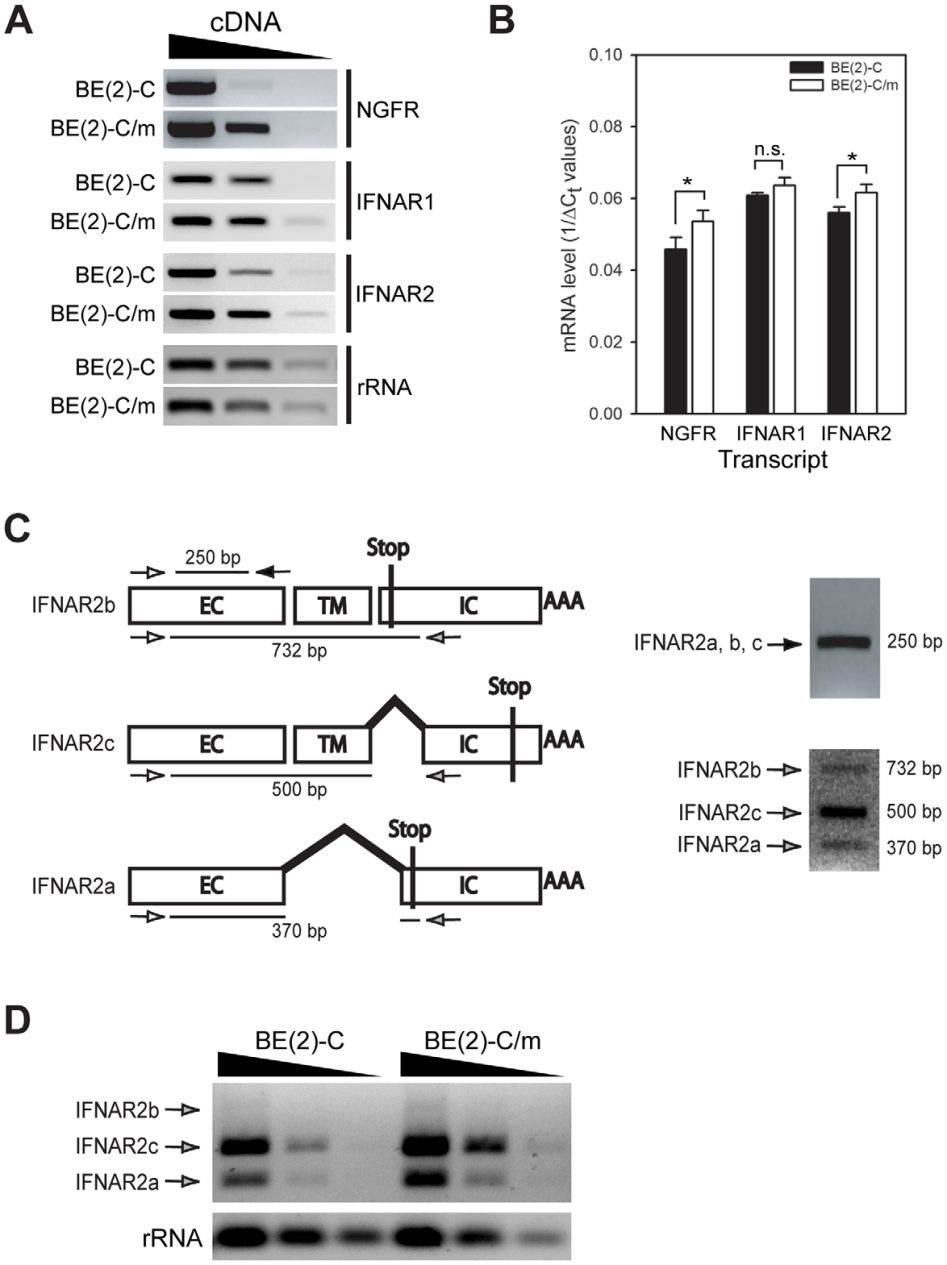
IFNAR mRNA analysis in undifferentiated BE(2)-C and differentiated BE(2)-C/m human neuronal cells. (A) Semi-quantitative RT-PCR analysis of total NGFR, IFNAR1, and IFNAR2 mRNA transcript expression. Ribosomal RNA (rRNA) was analyzed as a loading control and ten-fold serial dilutions of cDNA were used for PCR. (B) Quantitative RT-PCR analysis of NGFR, IFNAR1, and IFNAR2 mRNA transcript expression. Results are presented as inverse rRNA-normalized threshold cycle (1/ΔC_t_) values so that increased mRNA levels are associated with increased bar height. n.s., not significant; *p<0.05. (C) Schematic of IFNAR2 total (white and black arrows) and isoform-specific (white and grey arrows) primer design. (D) Semi-quantitative RT-PCR analysis of isoform-specific IFNAR2 mRNA transcript expression. Ten-fold serial dilutions of cDNA from the indicated cell line were used for PCR. Representative results from one of four independent experiments are shown.

We further examined IFNAR mRNA expression by RT-PCR (Fig. 2). Consistent with the flow cytometry results, total IFNAR2 mRNA was upregulated with neuronal differentiation, similar to the positive control nerve growth factor receptor (NGFR) mRNA, whereas we detected no significant increase in IFNAR1 mRNA accumulation by semi-quantitative RT-PCR (Fig. 2A). We further analyzed receptor transcript levels by qRT-PCR, which demonstrated that neuronal differentiation increased NGFR and IFNAR2 mRNA levels by approximately 16-fold and 4-fold, respectively, whereas no significant increase was seen in IFNAR1 mRNA levels (Fig. 2B). Transcription of the human IFNAR2 gene produces multiple variants that are derived from exon skipping, alternative splicing, and differential usage of a polyadenylation site, which when translated produce three distinct protein isoforms designated IFNAR2a, b, and c [34]. IFNAR2c is the signaling competent isoform [35], whereas IFNAR2b is a potential negative regulator [36] and IFNAR2a has been shown to have both agonistic and antagonistic properties [37]. We designed a single set of PCR primers to differentiate these three isoforms by semiquantitative RT-PCR (Fig. 2C). Although the IFNAR2b isoform did not amplify well, there were notable increases in both the IFNAR2a isoform and the signaling-competent IFNAR2c transmembrane isoform with neuronal differentiation (Fig. 2D).

**Figure 3:**
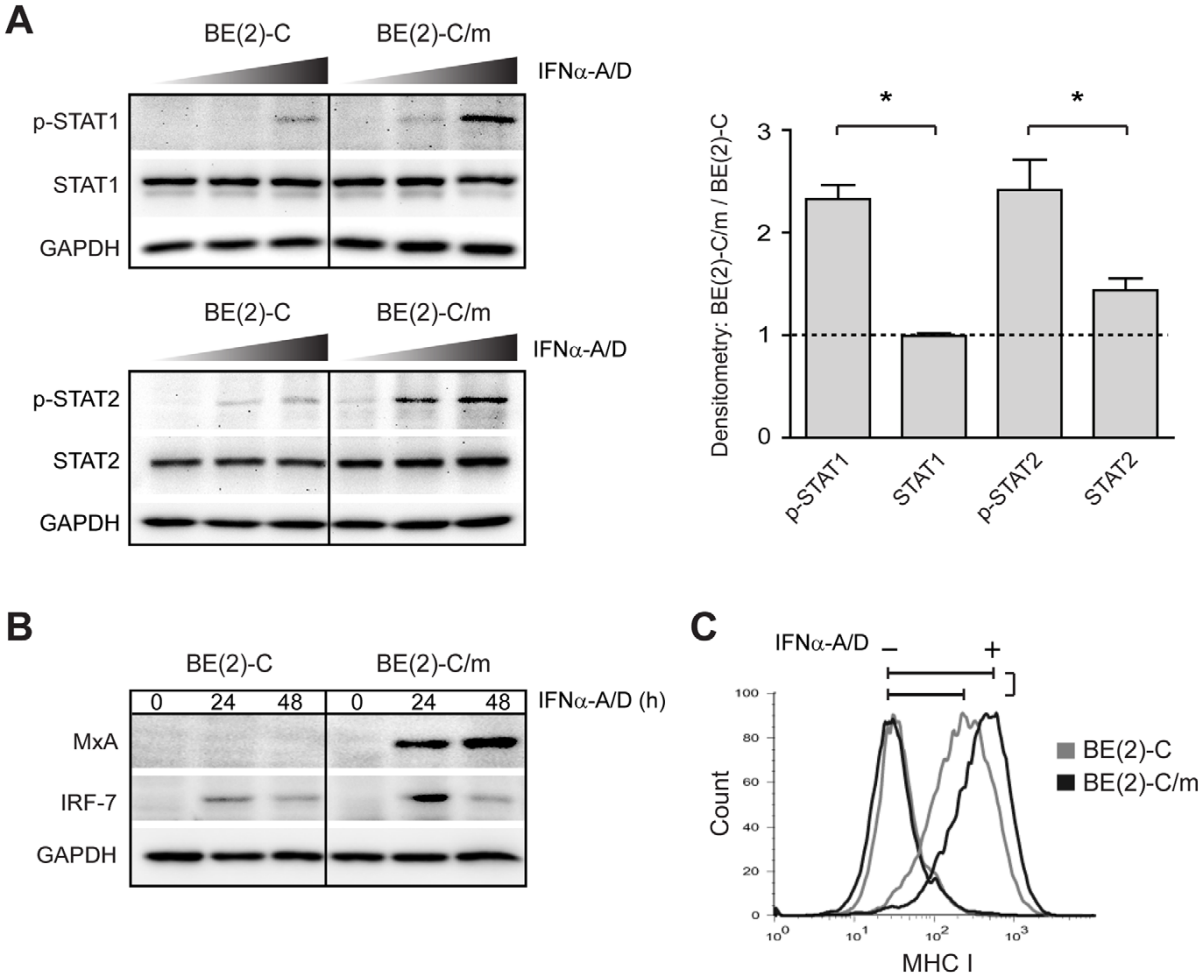
Differentiation-dependent changes in type I IFN pathway function in human BE(2)-C neuronal cells. (A) Immunoblot analysis of type I IFN-induced STAT phosphorylation. Cells were treated with 5, 50, or 500 U/mL IFNα-A/D and lysates were harvested and analyzed 30 min after stimulation for phosphorylated STAT1 (p-STAT1), total STAT1, phosphorylated STAT2 (p-STAT2), and total STAT2. Representative blots from one of three independent experiments are shown, and quantitative immunoblot results shown on the graph represent mean ± SEM densitometry ratios of differentiated over undifferentiated cells treated with 500 U/ml IFNα-A/D. *p<0.05. (B) Immunoblot analysis of type I IFN-induced MxA and IRF-7 expression. Cells were treated with 500 U/mL IFNα-A/D and harvested at the indicated times post-stimulation. Representative blots from one of three independent experiments are shown. (C) Flow cytometry analysis of type I IFN-induced surface MHC class I expression. Cells were unstimulated (−) or stimulated with 50 U/mL IFNα-A/D for 48 h (+). Representative overlaid histograms from one of four independent experiments are shown.

To examine type I IFN pathway function we analyzed IFNAR-dependent phosphorylation of the transcription factors STAT1 and STAT2 and IFN-stimulated gene induction (Fig. 3). Differentiated BE(2)-C/m cells had a two- to three-fold increase in STAT1 and STAT2 phosphorylation after stimulation with 500 U/ml IFNα-A/D, the highest concentration tested, but also had increased responsiveness at lower concentrations (Fig. 3A). We further analyzed downstream pathway activation by examining the induction of MxA, a direct antiviral effector [38], and IRF-7, a component of the viral pattern recognition receptor pathway [39], by immunoblot analysis (Fig. 3B), and also surface expression of the adaptive immune system component MHC class I by flow cytometry (Fig. 3C). Proteins encoded by all three genes showed increased expression 24 to 48 h after IFNα-A/D stimulation in differentiated BE(2)-C/m cells. The quantitative ratio of type IFN-stimulated MxA and IRF-7 expression after 24 h between differentiated and undifferentiated cells, determined by densitometry, were 7.4±0.9 and 2.7±0.4, respectively (*p*<0.05 relative to GAPDH; Fig. 3B). In addition, the fold increase in type I IFN-stimulated MHC class I expression, determined by median fluorescence intensity and represented by the brackets in Fig. 3C, was 12.9±0.9 and 6.3±0.5 for differentiated BE(2)-C/m and undifferentiated BE(2)-C cells, respectively (*p*<0.01). Taken together, these results indicated that human neuronal differentiation, modeled in BE(2)-C cells, was associated with selective increases in type I IFN pathway component expression and heightened cytokine responsiveness.

### Differentiation-dependent changes in neuronal type I IFN pathway function are recapitulated with overexpression of IFNAR2 and STAT2

**Figure 4:**
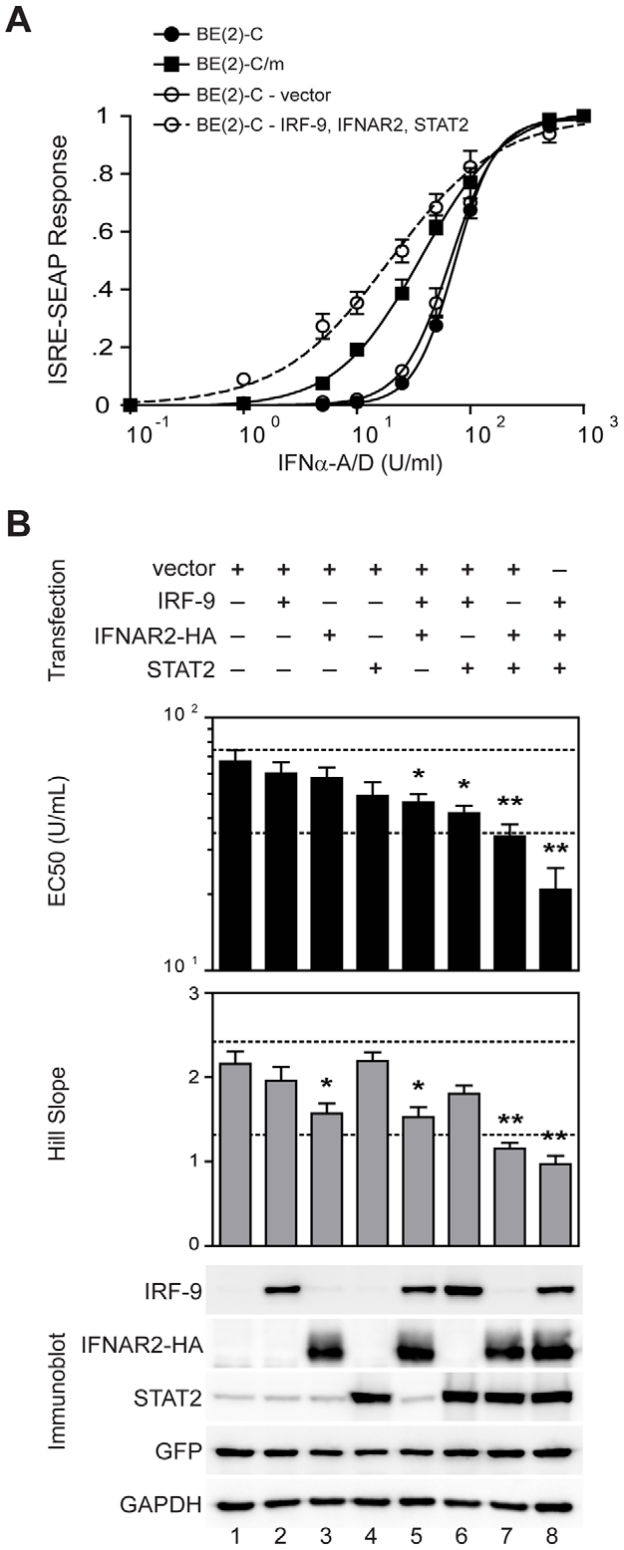
Overexpression of STAT2 and IFNAR2 is sufficient to recapitulate differentiation-dependent changes in neuronal type I IFN-stimulated gene expression. (A) Type I IFN dose-response curves were analyzed in undifferentiated BE(2)-C (closed circles), differentiated BE(2)-C/m (closed squares), and undifferentiated cells transfected with either empty vector (open circles, solid line) or IRF-9-, IFNAR2-, and STAT2-overexpression vectors (open circles, dashed line). Normalized SEAP responses 24 h after stimulation with IFNα-A/D were fit using a variable slope nonlinear regression to calculate EC_50_ and Hill slope values. (B) BE(2)-C cells were transfected with the indicated expression plasmid combinations, and EC_50_ and Hill slope values were calculated from independently fit type I IFN dose-response curves as noted above in (A). *p<0.05, **p<0.005, relative to vector control. Upper and lower dashed reference lines indicate calculated response values for untransfected undifferentiated and differentiated cells, respectively. Representative immunoblots from one of four independent experiments indicate equivalent overexpression of IRF-9, IFNAR2, and STAT2 in the appropriate samples. A GFP-overexpression plasmid was co-transfected as a control for transfection efficiency, and GAPDH was analyzed as a loading control.

We focused functional studies on the three canonical type I IFN signaling components that demonstrated differentiation-dependent expression in BE(2)-C/m cells: IRF-9, IFNAR2, and STAT2. We initially used loss-of-function experiments via stable shRNA-mediated suppression of pathway components, and found that IRF-9 knockdown reduced type I IFN stimulated gene activation in both immature BE(2)-C and mature BE(2)-C/m cells to similar levels (data not shown), suggesting that IRF-9 was necessary for neuronal type I IFN responses. However, we were only able to obtain approximately 40% suppression of basal IRF-9 expression levels, which limited interpretation of the functional significance of increased IRF-9 levels in BE(2)-C/m cells. Furthermore, initial experiments indicated that shRNA-mediated knockdown of STAT2 prevented differentiation of BE(2)-C cells (data not shown), thus precluding the full interrogation of canonical type I IFN signaling components via loss-of-function experiments. As an alternative and potentially more informative approach, we used gain-of-function experiments in BE(2)-C cells to directly examine which pathway components, when upregulated, were sufficient to overcome reduced responsiveness in undifferentiated cells (Fig. 4). To facilitate these studies, we generated BE(2)-C cells that stably expressed a tetracycline-regulated induction vector (Tet-ON) to conditionally induce the expression of IRF-9, IFNAR2, or STAT2. In addition, we stably introduced an ISRE-driven SEAP reporter gene to conveniently measure type I IFN-induced gene expression. We have previously demonstrated differentiation-dependent IFNα-A/D dose-response curves in BE(2)-C cells [6]. The Tet-ON/ISRE-SEAP cells showed a similar phenotype, where neuronal differentiation was associated with an approximate 50% decrease in EC_50_ (75.2±5.6 vs. 35.9±4.8 U/ml) and a downward shift in Hill slope (2.4±0.2 vs. 1.3±0.1) in favor of mass action binding (Fig. 4A, compare filled symbols). We initially examined whether overexpression of all three type I IFN signaling components in undifferentiated BE(2)-C cells would similarly alter the dose-response curve. While transfection of empty vector had no significant effect on IFNα-A/D responses, overexpression of IRF-9, IFNAR2, and STAT2 recapitulated the shift in both EC_50_ and Hill slope observed with neuronal differentiation (Fig. 4A, compare open symbols).

To determine the minimal components necessary to recapitulate the mature neuronal response to type I IFN stimulation, we performed detailed overexpression studies with individual components and all combinations thereof (Fig. 4B). Overexpression of individual components did not have a significant impact on either EC_50_ or Hill slope, with the exception of a reduction in Hill slope with IFNAR2 overexpression (Fig. 4B, lane 3). Overexpression of both IFNAR2 and IRF-9 significantly reduced EC_50_ and Hill slope (Fig. 4B, lane 5), whereas overexpression of both STAT2 and IRF-9 significantly reduced EC_50_ but not Hill slope (Fig. 4B, lane 6). Combined overexpression of IFNAR2 and STAT2 was sufficient to achieve significant shifts in both parameters of type I IFN responsiveness that were similar to those seen with differentiation (Fig. 4B, lane 7, compare to lower dashed reference lines). Although the trend with addition of IRF-9 overexpression was a further reduction in both EC_50_ and Hill slope (Fig. 4B, lane 8), this increased reduction was not statistically significant when compared to overexpression of only IFNAR2 and STAT2. These results indicated that overexpression of IFNAR2 and STAT2 in BE(2)-C cells was sufficient to recapitulate the heightened type I IFN-stimulated gene expression observed in differentiated neuronal cells.

**Figure 5:**
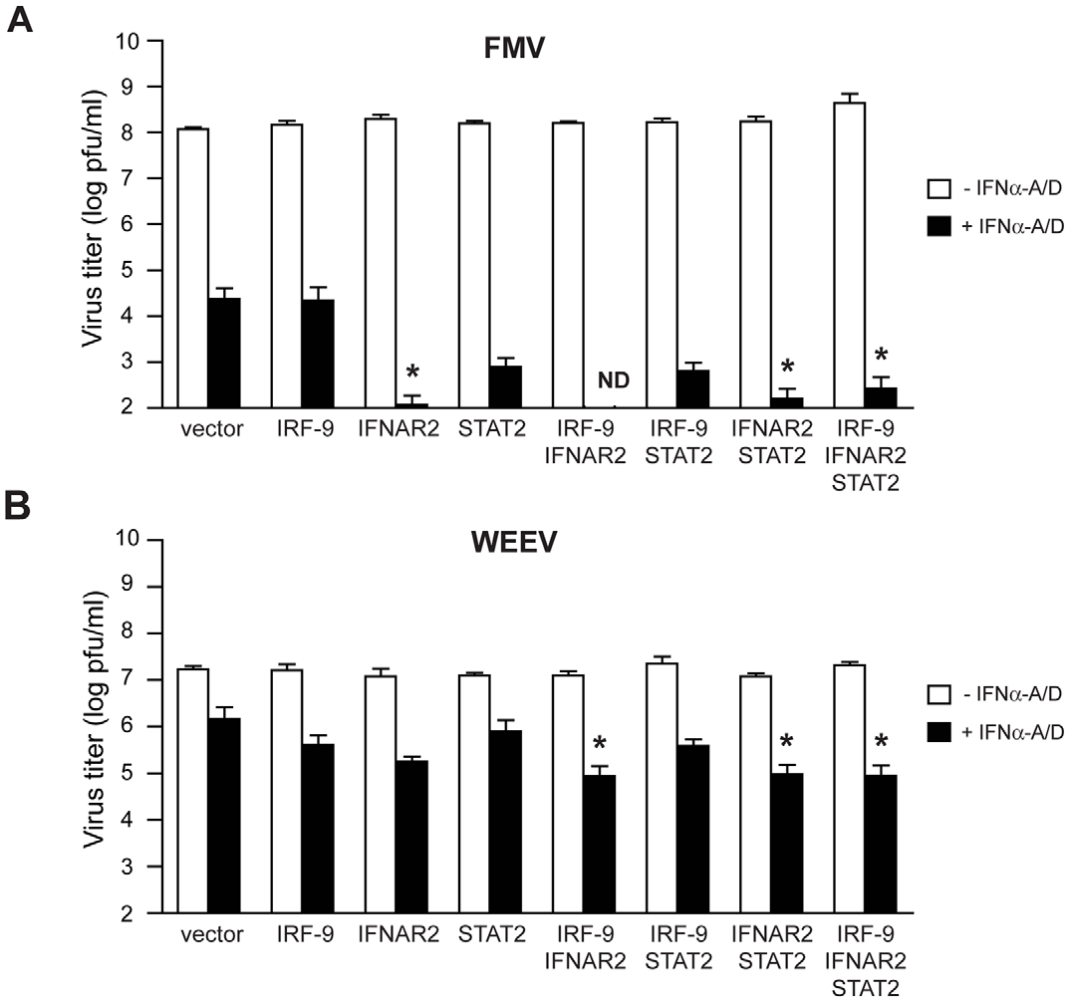
Overexpression of type I IFN signaling components selectively modulates antiviral responses in human neuronal cells. Undifferentiated BE(2)-C cells were transfected with the indicated expression vector combinations and either unstimulated (open bars) or stimulated with 500 U/mL IFNα-A/D for 24 h (closed bars), infected with the indicated virus at an MOI of 0.01, and infectious virus titers in tissue culture supernatants were measured by plaque assay at 72 hpi for FMV (A) or 36 hpi for WEEV (B). Results represent mean ± SEM from at least four independent experiments, and the change in virus titer with IFNα-A/D stimulation was used for statistical analyses. ND, not detected. *p<0.05, relative to the IFN-stimulated reduction in vector control-transfected cells.

To determine the impact of individual and combined exogenous expression of IRF-9, IFNAR2, and STAT2 on virus replication in BE(2)-C cells, we challenged transfected cells with two neurotropic alphaviruses (Fig. 5). We chose related pathogens that display different levels of intrinsic virulence: the CM4-146 strain of FMV, a low virulence member of the WEEV complex that can be safely handled under routine BSL-2 conditions [40], and the Cba-87 strain of WEEV, an epizootic isolate that is highly virulent in both rodents [40], [41] and primates [42] and requires BSL-3 containment conditions. We have previously demonstrated that human BE(2)-C cells have differentiation-dependent responses to WEEV infection [6], and preliminary experiments showed that FMV infection produced a similar differentiation-dependent phenotype (data not shown). IFNα-A/D priming of control BE(2)-C cells transfected with empty vector reduced FMV titers by almost four logs, and this reduction was accentuated by approximately 100-fold with overexpression of IFNAR2 alone or in any combination with IRF-9 and/or STAT2 (Fig. 5A). In contrast, IFNα-A/D priming reduced WEEV titers in control cells by approximately one log, and this reduction was only accentuated when IFNAR2 was overexpressed in combination with either IRF-9 or STAT2, or when all three components were expressed together (Fig. 5B). However, the level of reduction with IFNα-A/D priming never exceeded two to three logs with any signaling component combination in BE(2)-C cells, which was consistent with previously observed type I IFN-mediated reduction of virus titers in differentiated BE(2)-C/m cells infected with WEEV [6]. These results were also consistent with the IRSE promoter-driven reporter gene studies (Fig. 4). Although it is tempting to speculate that lower virulence organisms such as FMV may be more susceptible to enhanced neuronal innate immune responses compared to more virulent pathogens such as WEEV, differences in experimental conditions and the in vitro nature of our approach preclude drawing such definitive conclusions. Nevertheless, taken together these results suggested that increased IFNAR2 expression was an important determinant of enhanced type I IFN responses and cell-autonomous control of both FMV and WEEV replication in mature neurons.

### Enriched populations of neural progenitor cells (NPCs) and mature neurons can be derived from hESCs

**Figure 6:**
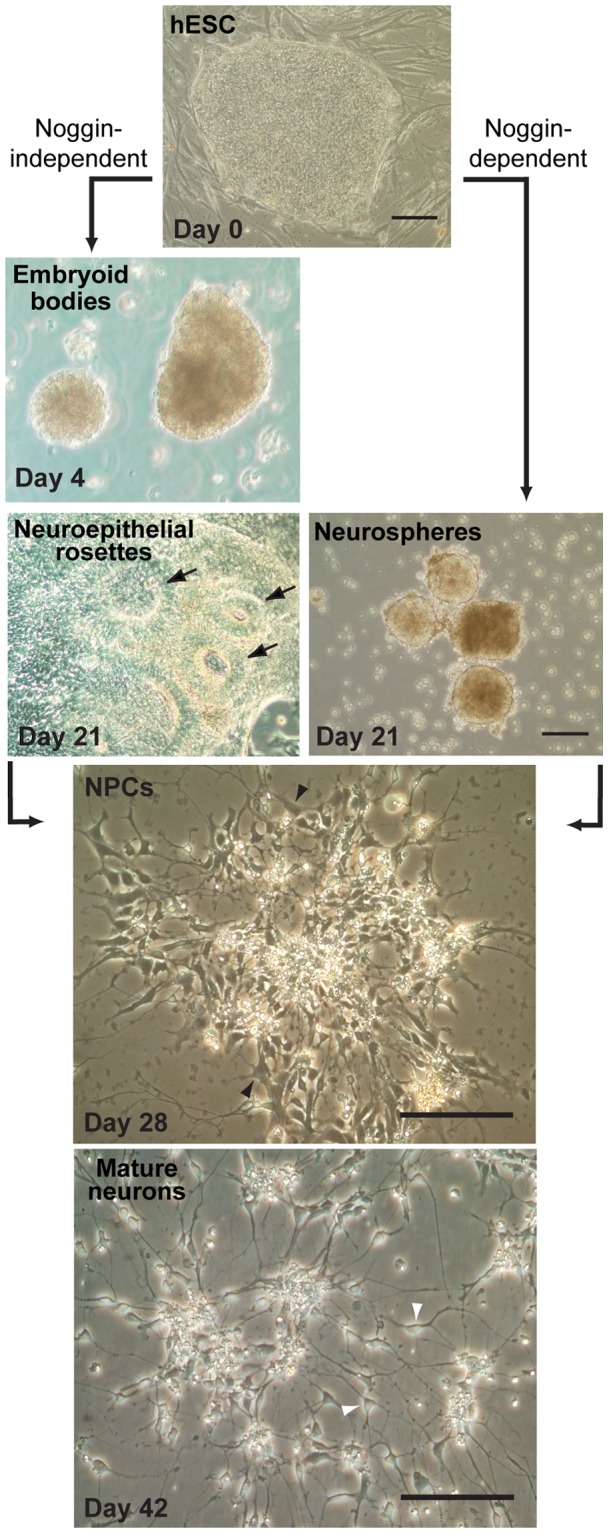
Differentiation of hESC to NPCs and mature neurons. Phase-contrast micrographs of a pluripotent hESC colony growing on a layer of irradiated mouse embryonic fibroblasts on day 0 (top image), subsequent differentiation in the absence of noggin into embryoid bodies on day 4 and neuroepithelial rosettes on day 21 (left images) or in the presence of noggin into neurospheres in suspension culture on day 21 (right image), and final differentiation into adherent cultures enriched in NPCs on day 28 and mature neurons by day 42 (bottom images). Arrows in day 21 noggin-independent cultures indicate neuroepithelial rosettes. Open arrowheads in day 28 NPC cultures indicate characteristic cells with large perikaryon and minimal processes, whereas closed arrowhead in day 42 mature neuron cultures indicate characteristic cells with small perikaryon and extensive processes. Scale bars  = 200 μm.

Immortalized cell lines provide reliable and reproducible resources to model neuronal maturation in culture, and have been used extensively to investigate differentiation-dependent changes that impact immune system function and responses to various neurotropic viruses [6], [15], [16]. However, immortalized cell lines have potential drawbacks, including the inability to fully reproduce the physiologic responses of primary cells. To validate select neuronal differentiation-dependent changes in innate immunity observed in BE(2)-C cells using non-malignant human cells, we modified established methods of hESC differentiation [28], [29], [43], [44], [45] to develop protocols that produced pure neural-lineage cells along the spectrum of differentiation from NPCs to mature neurons (Fig. 6). The first protocol allowed hESCs to free-form differentiate into cystic embryoid bodies by day 4, which were subsequently plated and expanded to produce neuroepithelial rosettes by day 21 (Fig. 6, noggin-independent protocol, left images). The second protocol used the bone morphogenic protein antagonist noggin to induce hESC differentiation directly into neurospheres by day 21 (Fig. 6, noggin-dependent protocol, right image). For both protocols, NPCs were subsequently generated by selective plating conditions in defined growth media to produce cells that morphologically resembled NPCs by day 28, with moderately-sized perikarya and small, largely unbranched neurites, and cells that morphologically resembled mature neurons by day 42, with small perikarya and an extensive network of branched processes (Fig. 6, bottom two images). Both noggin-independent and noggin-dependent protocols reproducibly generated NPCs, although the latter protocol was less labor-intensive and routinely resulted in higher yields and purity.

**Figure 7:**
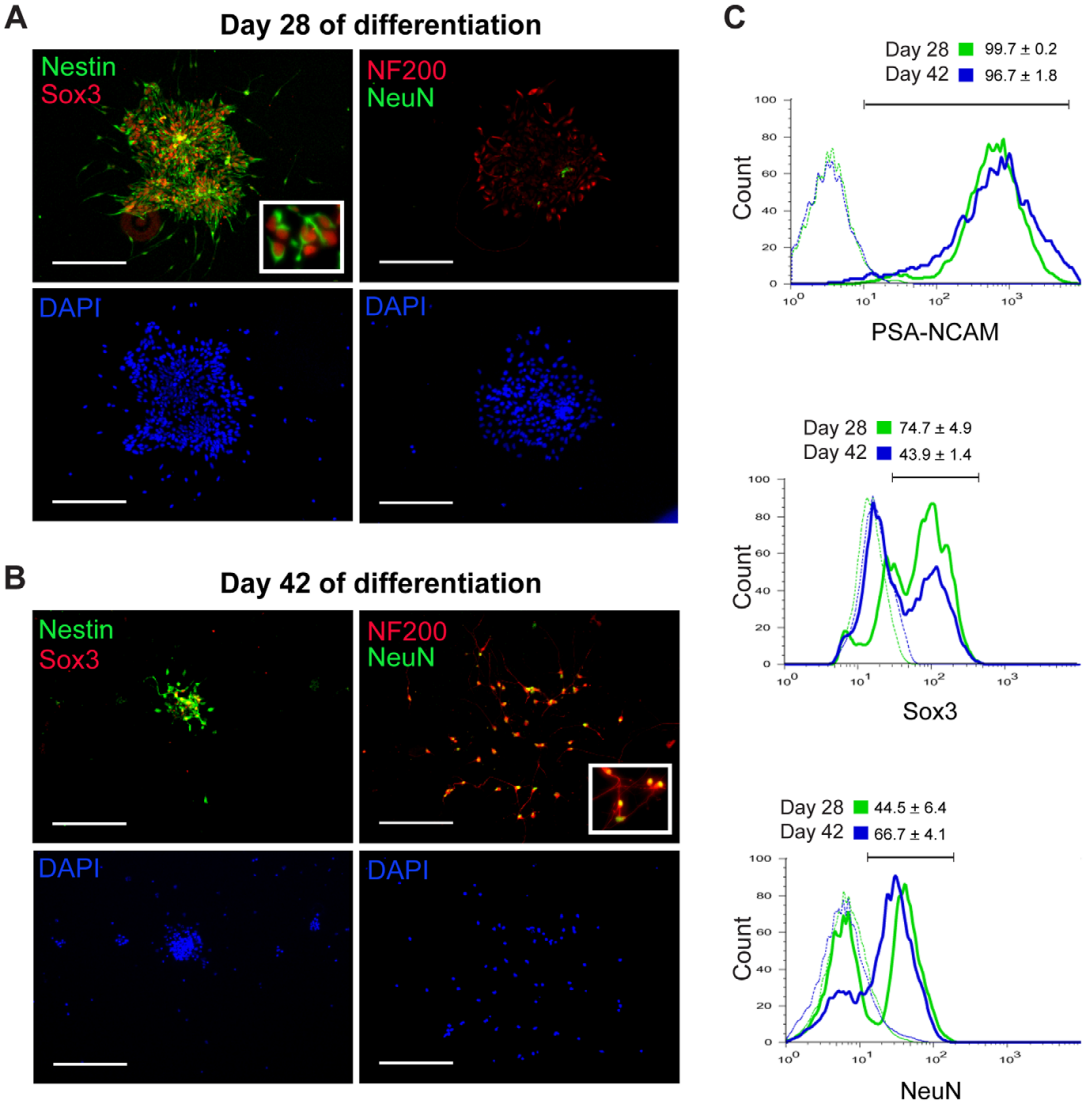
hESC-derived cultures are neural-lineage pure and enriched in NPCs at day 28 or mature neurons at day 42. Immunocytochemical analysis of day 28 differentiated (A) and day 42 differentiated (B) cultures generated using the noggin-dependent protocol outlined in Fig. 6. Expression of the indicated marker is shown as an overlay image (top panels), and nuclear DAPI staining identifies all cells in the field (bottom panels). Scale bars  = 200 μm. Higher magnification inset images more clearly depict cell morphology and intracellular staining pattern. (C) Flow cytometry analysis of day 28 differentiated (green lines) and day 42 differentiated (blue lines) neuronal cultures derived using the noggin-dependent protocol outlined in Fig. 6. Surface PSA-NCAM expression assessed neural-lineage purity. Intracellular SOX3 and NeuN expression were used to approximate NPC and mature neuronal percentages, respectively. Representative overlaid histograms from one of two independent experiments are shown, where quantitative values represent mean ± SD percentage positive relative to isotype control. The noggin-dependent differentiation protocol produced less pure populations of NPCs and mature neurons, where NPC cultures were ∼43% SOX3+ and mature neuronal cultures were ∼58% NeuN+ (data not shown).

We characterized NPCs and mature neurons by both immunocytochemistry and flow cytometry (Fig. 7). At day 28 of differentiation, cells prominently expressed the transcription factor Sox3 and the intermediate filament protein nestin (Fig. 7A, left panel), which are markers of undifferentiated NPCs [46], [47]. In contrast, expression of NeuN, a transcription factor associated with mature neurons [48], and NF200, a heavy neurofilament protein also associated with mature neurons [49], was confined to the culture perimeter (Fig. 7A, right panel), consistent with previously described radial differentiation of hESC-derived NPCs in culture [28]. Culture purity at day 28 of differentiation assessed by flow cytometry showed that >99% of cells expressed the pan-neural lineage marker PSA-NCAM (Fig. 7C, green lines). The majority of cells (∼75%) also expressed Sox3, which indicated a predominance of NPCs, whereas a minority of cells (∼45%) expressed NeuN. At day 42 of differentiation, cells showed prominent expression of the mature neuronal markers NeuN and NF200 (Fig. 7B, right panel). In contrast, Sox3 expression was much less prominent and nestin expression was primarily restricted to densely clustered cells containing residual NPCs (Fig. 7B, left panel). Flow cytometry demonstrated that >96% of the day 42 culture cells expressed PSA-NCAM (Fig. 7C, blue lines), but in contrast to day 28 NPC cultures, the majority of cells (∼67%) expressed NeuN and the minority (∼44%) expressed Sox3, which suggested a predominance of mature neurons. We observed no glial fibrillary acidic protein expression in day 42 differentiated cultures (data not shown), which indicated the absence of astrocyte contamination. These results demonstrated that pure populations of human neural lineage-restricted cells, enriched in NPCs and mature neurons at day 28 and 42 of differentiation, respectively, could be reliably generated from hESCs for subsequent immunological and virological analyses.

### Differentiation of human NPCs to mature neurons enhances type I IFN pathway component expression and function

**Figure 8:**
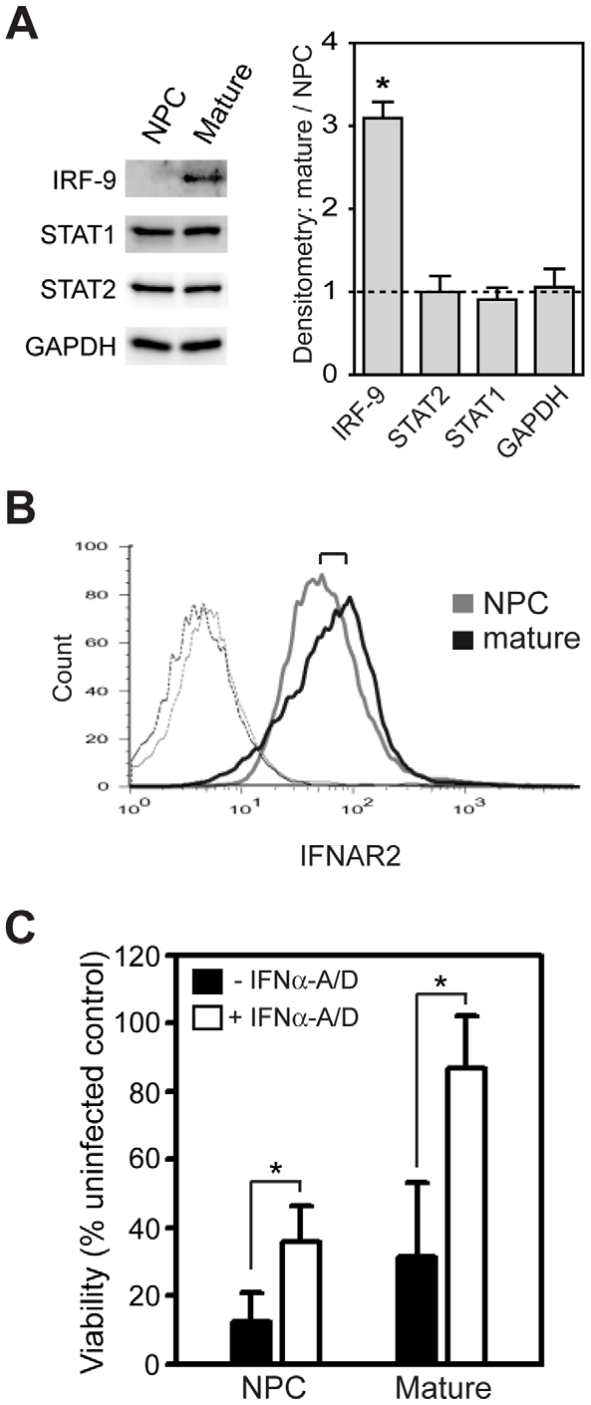
Differentiation of hESC-derived neurons enhances type I IFN pathway component expression and function. (A) Lysates from hESC-derived NPCs and mature neurons were immunoblotted for basal IRF-9, STAT1, and STAT2 expression. GAPDH was analyzed as loading control. Representative blots from one of three independent experiments are shown, and quantitative immunoblot results shown on the graph represent mean ± SEM densitometry ratios of mature versus immature cells. *p<0.05, relative to GAPDH. (B) Flow cytometry analysis of basal surface IFNAR2 expression on hESC-derived neurons. Solid grey and black lines represent IFNAR2 levels on immature and mature cells, respectively. Dashed lines indicate background fluorescence levels obtained with isotype-matched control antibodies. Representative overlaid histograms from one of four independent experiments are shown. (C) Cell viability analysis of type I IFN-mediated protection from WEEV-induced cell death. hESC-derived NPCs or mature neurons were either unprimed (closed bars) or primed with 50 U/mL IFNα-A/D (open bars) for 24 h prior to infection with WEEV at an MOI of 0.1, and viability was analyzed at 72 hpi. Results represent mean ± SEM from four independent experiments (*p<0.05; n = 2 each for noggin-dependent and noggin-independent protocols).

To determine whether hESC-derived neurons displayed differentiation-dependent changes in intrinsic innate immune system component expression and function similar to BE(2)-C cells, we initially examined STAT1, STAT2, IRF-9, and IFNAR levels in hESC-derived NPCs and mature neurons (Fig. 8). Immunoblot analysis revealed an approximate three-fold increase in IRF-9 expression in hESC-derived mature neurons compared to NPCs, but no significant differences in basal expression of STAT1 or STAT2 (Fig. 8A). In addition, we observed an approximate 50% increase in surface IFNAR2 expression in mature hESC-derived neurons compared to NPCs, where the quantitative ratio of IFNAR2 expression between mature and immature cells, determined by median fluorescence intensity and represented by the bracket in Fig. 8B, was 1.6±0.2 (*p*<0.05). These results were consistent with those obtained with BE(2)-C cells (see Fig. 1), and indicated that at least two type I IFN signaling pathway components, IRF-9 and IFNAR2, were upregulated with differentiation of both hESC- and BE(2)-C-derived neurons.

To determine whether altered type I IFN pathway component expression influenced cell-autonomous responses to virus infection in hESC-derived neurons, we challenged both NPCs and mature neurons with WEEV either in the presence or absence of type I IFN priming (Fig. 8C). WEEV is highly cytopathic in most cultured cell lines, and one prominent characteristic of cellular differentiation-dependent changes in BE(2)-C neuronal cells is a heightened protective response to type I IFN exposure that results in enhanced cell survival after infection [6]. In the absence of IFNα-A/D priming both NPCs and mature neurons derived from hESCs were susceptible to virus-induced cell death (Fig. 8C, closed bars). However, although 24 h priming with IFNα-A/D increased cell survival after WEEV infection for both NPCs and mature neurons (Fig. 8C, open bars), the type I IFN-mediated increase in cell survival was approximately two-fold greater for hESC-derived mature neurons (55.3±14.2%) compared to NPCs (23.4±7.5%; *p*<0.05) (Fig. 8C, compare open and closed bars), which was similar to the differentiation-dependent increase in type I IFN-mediated cytoprotective responses observed with BE(2)-C-derived neuronal cells infected with WEEV [6]. Taken together, these results indicated that differentiation of hESC-derived neural progenitors to mature neurons was accompanied by heightened type I IFN pathway activity that resulted in increased protection against neurotropic alphavirus infection.

## Discussion

Neuronal development is a complex, dynamic, and incompletely understood process. In addition to traditional neurophysiologic changes that occur, such as axonal outgrowth and synapse formation, other crucial physiologic changes also likely exist, including modulation of innate immune function. We utilized a previously established neuronal cell line model [6] in conjunction with a versatile culture system to reliably generate enriched populations of NPCs and mature neurons from hESCs to examine the effects of human neuronal differentiation on innate immune pathway function and susceptibility to neurotropic alphaviruses. We drew three main conclusions from these studies: (i) the canonical type I IFN signaling pathway components IRF-9 and IFNAR2 were upregulated with neuronal differentiation; (ii) neuronal differentiation was associated with increased functional responses to type I IFNs; and (iii) IFNAR2 expression was an important determinant of neuronal differentiation-dependent responses. These findings suggest that human immature neurons and NPCs may be more susceptible to viral infection and virus-induced cell damage compared to mature neurons due in part to reduced type I IFN-mediated innate immune system function.

Although hESCs are frequently viewed as potential precursors for cell replacement therapy in patients [50], [51], [52], the pluripotent capacity of these cells can also be exploited in experimental studies to examine cell type-specific physiologic processes [53]. We chose to use hESC-derived cells at two distinct stages of neural differentiation, NPCs and mature neurons, to validate several key features of innate immunity and WEEV-induced cell damage initially identified with the BE(2)-C model system. However, hESC-derived neuronal culture systems also have potential broad applicability for detailed pathogenesis studies with WEEV and other clinically important neurotropic viruses. Indeed, several recent studies have used hESC-based culture systems similar to the one described in this report to investigate borna disease virus, varicella zoster virus, and herpes simplex virus [24], [54], [55], although none of these studies specifically examined innate immune system function in the context of viral infection, but rather focused mainly on the stage of neuronal development at which cells were most permissive to viral infection. Furthermore, hESC culture systems can also be used to generate and examine mature neuronal subtypes [56]. Preliminary experiments revealed several mature neuronal subtypes in day 42 differentiated cultures, including GABAergic, glutamatergic, and dopaminergic cells (J. Farmer and D. Miller, unpublished data). The ability to selectively drive the maturation of hESC-derived neurons toward a particular subtype would have substantial implications for the study of viral pathogenesis, as several neurotropic viruses have selective neurotropism within the CNS that can have a significant impact on disease presentation and outcome. For example, Japanese encephalitis virus causes Parkinson's disease-like symptoms by targeting the basal ganglia [57], [58], an area highly enriched in GABAergic neurons. Finally, while we focused primarily on differentiation-dependent changes in innate immunity that influenced susceptibility to virus-induced damage, the model of human neural differentiation presented in this report and others [24], [54], [55] are readily amenable to studies involving non-infectious inflammatory conditions whose proposed underlying pathophysiology involves innate immunity, such as amyotrophic lateral sclerosis [59].

The demonstration that neuronal differentiation-dependent IFNAR2 upregulation was an important determinant of enhanced type I IFN responsiveness and reduced viral susceptibility is consistent with previous observations of maturation-dependent IFNAR upregulation in human monocytes [60] and cell-type specific differences in IFNAR2 expression that correlate with type I IFN-dependent viral inhibition in cardiac fibroblasts and myocytes [61]. We also observed a significant upregulation of IRF-9 with neuronal differentiation, but overexpression of this canonical pathway component had only minimal impact on type I IFN responses. This suggested that either IRF-9 was not a limiting factor or that type I IFN signal transduction in human neuronal cells was IRF-9-independent, which has been described for other cell types [62]. As noted above, preliminary studies indicated that shRNA-mediated knockdown of IRF-9 in BE(2)-C cells suppressed IFNα-A/D stimulated gene activation (data not shown), suggesting that IRF-9 may be required but not limiting in neuronal type I IFN responses. We did not specifically explore the impact of neuronal differentiation on non-canonical type I IFN signaling pathways, such as those mediated by NF-κB [63]. Thus, the full contribution of individual components within the complex signaling cascades that result from type I IFN stimulation in neurons remains unknown and a potentially productive area for future studies.

The type I IFN pathway is an essential component of the innate immune response to neurotropic viral infections [7], [9], [10], but its function is not solely limited to antiviral defense. The human genome encodes 17 distinct type I IFNs: 12 IFNα subtypes and single IFNβ, ε, κ, ω, and ν proteins [64]. All of these type I IFNs exert their effects through a common receptor composed of IFNAR1 and IFNAR2 subunits [65]. In the current study we used primarily a universal hybrid type I IFN [27], but we have previously demonstrated that human neuronal cells show preferential responsiveness to distinct type I IFNs in the activation of cellular antiviral pathways [6]. Diverse and subtype-specific cellular responses to unique type I IFNs have been described, including activation of pro-apoptotic genes, repression of anti-apoptotic genes, modulation of cellular differentiation, and repression of angiogenesis [64]. The physiologic significance of neuronal differentiation-dependent responsiveness on these processes is currently unknown. However, IFNβ has been shown to decrease the proliferation and maturation potential of murine neuronal precursors [66], and unregulated expression of IFNα within the CNS results in premature neurodegeneration [67]. These observations suggest that modulation of type I IFN responses via altered IFNAR2 expression may be a crucial regulatory mechanism during neuronal differentiation that represents a balance between beneficial and potentially harmful cytokine-mediated effects.

The demonstration of neuronal differentiation-dependent type I IFN activity and virus susceptibility provides a plausible explanation for the clinical observation that several neurotropic arbovirus infections are particularly severe in pediatric patients [14]. Although systemic immune system maturation and function likely contribute to this observation, experimental studies with Sindbis and Semliki Forest viruses, which are model alphaviruses that produce age-dependent encephalitis in mice, provide compelling evidence that intrinsic, adaptive immune system-independent CNS changes play a prominent role [68], [69], [70], [71]. Our results suggest that modulation of neuronal type I IFN responsiveness represents a potentially important component of these intrinsic changes, where immature neurons or NPCs in pediatric patients may be more susceptible to virus-induced damage due to reduced innate immune responses, even in the setting of adequate systemic or local type I IFN production. Irreparable damage to these essential CNS cells may be, in part, responsible for long-term permanent neurological sequelae after viral infection [14]. Experimental small rodent models with WEEV and other virulent encephalitic arboviruses have been developed [72], and the availability of conditional cell-type specific transgene expression or deletion in mice [73] provide an opportunity to directly test this hypothesis in vivo.
